# Developing precision medicine for people of East Asian descent

**DOI:** 10.1186/s12929-016-0299-3

**Published:** 2016-11-11

**Authors:** Stacy L. McAllister, Katherine Sun, Eric R. Gross

**Affiliations:** Department of Anesthesiology, Perioperative and Pain Medicine, School of Medicine, Stanford University, 300 Pasteur Drive, Grant Building, Room S290, Stanford, CA 94305 USA

**Keywords:** Aldehyde, Acetaldehyde, ALDH2, Precision medicine, East Asian, Nitroglycerin, Esophageal, Cancer, Aldehyde dehydrogenase 2

## Abstract

The goal of precision medicine is to separate patient populations into groups to ultimately provide customized care tailored to patients. In terms of precision medicine, ~540 million people in the world have a genetic variant of the aldehyde dehydrogenase 2 (ALDH2) enzyme causing a flushing response and tachycardia after alcohol consumption. The genetic variant is identified as ALDH2*2 and originates from East Asian descendants of the Han Chinese. The variant is particularly important to consider when discussing lifestyle choices with patients in terms of risk for developing specific diseases, preventative screening, and selection of medications for treatment. Here we provide examples why patients with an ALDH2*2 variant need more individualized medical management which is becoming a more standard practice in the precision medicine era.

## Background

In 2015, the National Institutes of Health launched the precision medicine initiative. This initiative describes precision medicine as “an emerging approach for disease prevention and treatment taking into account people’s individual variations in genes, environment, and lifestyle” with the ultimate goal of implementing these concepts into clinical practice [1].

Some examples of precision medicine already exist in medical practice. For example in pediatrics, a genetic panel is typically used when a child is born, known as a newborn screen, which in California tests for 57 different conditions. If one of these genetic conditions is identified from the screen, individualized and rather specific care is provided which can prevent serious health problems. Another example is recommendations for disease surveillance based upon single gene variants which increase the risk for colon cancer as seen in individuals with Lynch syndrome or familial adenomatous polyposis. These patients have established personalized guidelines relevant to the frequency of medical surveillance for colon cancer by colonoscopy which differs from colon cancer screening measures recommended to the general population [2].

Physicians are now also considering the influence of genetic variants on drugs they prescribe when treating specific conditions. For example, a variant in P450 CYP2C19 reduces the amount of the active form of clopidogrel (a drug given when treating atrial fibrillation). This is concerning since a subset of people with atrial fibrillation and the CYP2C19 variant may not achieve a therapeutic level of clopidogrel if prescribed the medication. Recently, physicians have started implementing screening for the CYP2C19 variant as part of the medical decision making process when determining which drug to prescribe for atrial fibrillation [3].

Similar to the examples above, here we describe a genetic variant which when factored in with environment and lifestyle choices will influence medical management, preventative medicine, and drug treatment choices. This genetic variant of the aldehyde dehydrogenase 2 (ALDH2) enzyme is present in ~8 % of the world's population. Here, we also briefly outline the initial steps for implementing precision medicine in the clinic for patients with this variant.

## Main text

**Fig. 1:**
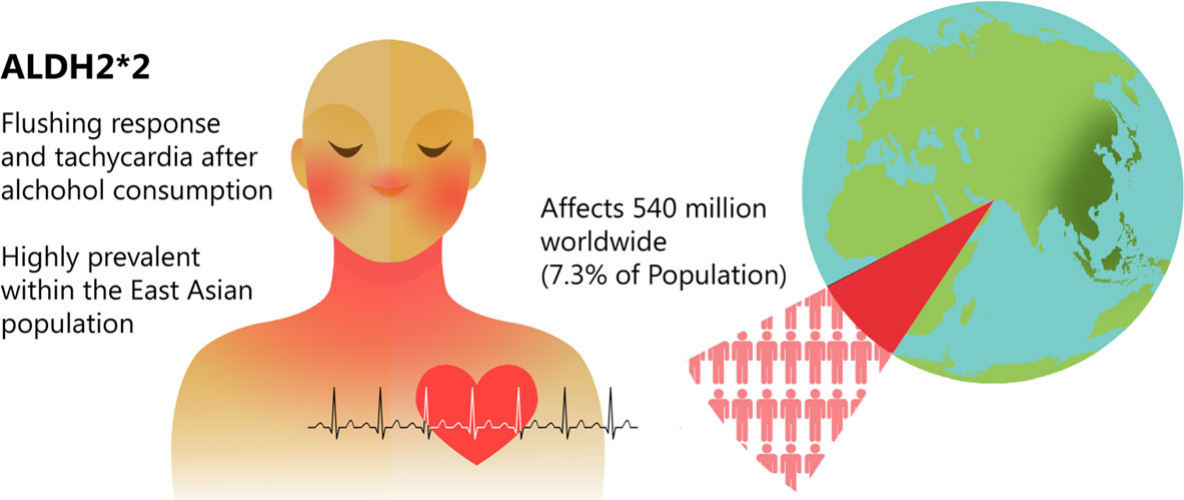
The genetic variant ALDH2*2. The ALDH2*2 variant is present in 540 million people in the world mainly of Han Chinese descent. The variant is identifiable by the phenotypic response of facial flushing after alcohol consumption. People heterozygotic for the genetic variant (ALDH2 *1*2) have at least a 60 % decrease in enzymatic activity while those homozygotic (ALDH2 *2*2) have greater than a 95 % decrease in enzymatic activity

Approximately 540 million people have a genetic variant of the aldehyde dehydrogenase 2 (ALDH2) enzyme which causes a flushing response and tachycardia after alcohol consumption (Fig. 1). The genetic variant, identified as ALDH2*2, is almost exclusively present in East Asian descendants of Han Chinese and is particularly important when considering risk factors for disease, decisions in medical treatment, and drug selection. Recently, extensive reviews have covered the molecular biology and importance of the ALDH2*2 variant in human health [4, 5]. Here, we concisely discuss 2 examples of how the ALDH2*2 variant factors into decisions regarding medical management and drug selection. We believe these two examples should be used as a foundation for developing a more defined precision medicine platform for those with an ALDH2*2 variant.

### Developing precision medicine for ALDH2*2 in relation to cancer risk

Undoubtedly, lifestyle choices contribute to the risk of developing cancer. In particular, alcohol consumption and cigarette smoking are two lifestyle choices which increase the risk for esophageal cancer. Cigarettes contain several reactive aldehydes (such as acrolein, formaldehyde and acetaldehyde) which are known carcinogens [6]. The acetaldehyde produced from breaking down alcohol also is metabolized to acetaldehyde by the ALDH2 enzyme.

**Fig. 2:**
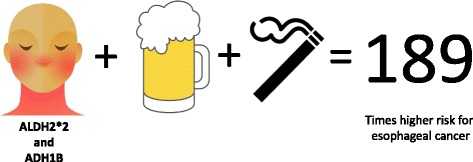
The influence of genetics and lifestyle choices on esophageal cancer risk. People with the ALDH2*2 variant who consume alcohol and are also fast metabolizers of alcohol by having the ADH1B variant have an increased risk for developing esophageal cancer. If the person also smokes cigarettes, the risk of developing esophageal cancer is potentially 189 times higher

In comparison to the United States, countries in Asia have a higher incidence of esophageal cancer [7]. Reactive aldehyde carcinogens are less efficiently metabolized by those with an ALDH2*2 variant. With this in mind, people with an ALDH2*2 variant are at a much greater risk for esophageal cancer. A strong association between alcohol consumption and increased esophageal cancer risk in the ALDH2*2 population has been established [8, 9]. Alarmingly, people with an ALDH2*2 variant and also an alcohol dehydrogenase variant (ADH1B, that causes a rapid conversion of alcohol to acetaldehyde) with a history of drinking alcohol and smoking cigarettes, have an odds ratio of 189 for developing esophageal cancer (Fig. 2) [10].

Taking this risk into consideration, similar to published outlined measures for gastrointestinal cancers with reference to specific genetic variants [2], increased consideration should be given to developing screening measures for esophageal cancer in the ALDH2*2 population. This could potentially reduce the incidence of esophageal cancer within the East Asian population. Further, screening for the ALDH2*2 gene could be used as topic of conversation with patients as a preventative measure to reduce esophageal cancer risk. These discussions could include how the ALDH2*2 variant in combination with lifestyle choices such as drinking alcohol or smoking cigarettes increases the relative risk of developing esophageal cancer. Together, effectively communicating these lifestyle risks can be a powerful means to reduce esophageal cancer incidence.

### Integrating the ALDH2*2 variant into medical decisions when prescribing drugs

**Fig. 3:**
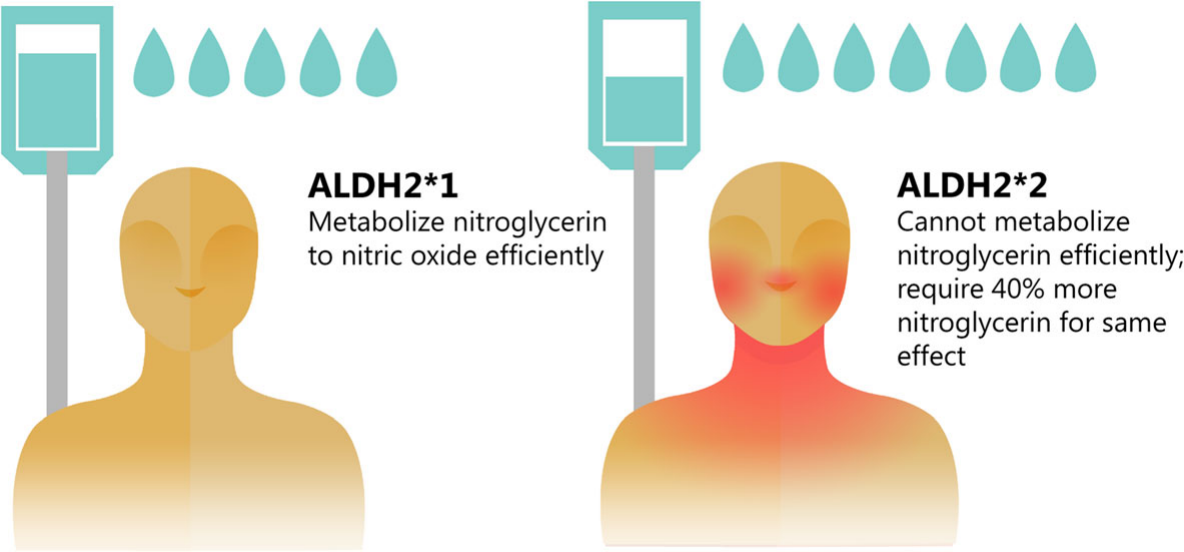
Reduced metabolism of nitroglycerin to nitric oxide. The esterase activity for the ALDH2 enzyme is required to convert nitroglycerin to nitric oxide. Those with the ALDH2*2 variant also have a reduction in esterase activity, which in turn causes nitroglycerin to be less efficiently metabolized to nitric oxide. Approximately 40 % more nitroglycerin may need to be given to people with the ALDH2*2 variant to achieve the same effect as those with the ALDH2*1 enzyme

Besides the importance for ALDH2 in metabolizing acetaldehyde (the breakdown product of alcohol), ALDH2 is also an important enzyme in drug metabolism. The efficacy of several drugs are impacted by the ALDH2*2 variant [4]. Specifically, ALDH2 metabolizes nitroglycerin to nitric oxide in order to cause blood vessel dilation. Importantly, those with an ALDH2*2 variant are less efficient at breaking down nitroglycerin to nitric oxide, effectively limiting the vasodilatory effect of nitroglycerin. The catalytic activity to metabolize nitroglycerin is 10-fold less for the ALDH2*2 enzyme compared to the ALDH2*1 enzyme [11]. Studies in East Asians, measuring forearm blood flow response to nitroglycerin show that ALDH2*2 variants have a 40 % reduced response compared to those without a *2 variant as measured by vasodilation (Fig. 3) [12].

Importantly, the influence of ALDH2*2 on nitroglycerin metabolism may impact medical decision making in several medical disciplines including anesthesiology, obstetrics, cardiology, internal medicine, and emergency medicine. Nitroglycerin is commonly given after cardiac bypass for a heart valve replacement or coronary artery bypass grafting. Nitroglycerin is also used in obstetrics to relax the uterus. In cardiology and emergency medicine, nitroglycerin is frequently given to relieve chest pain associated with myocardial ischemia. Many of these nitroglycerin applications in medical practice may need to consider potential ALDH2*2 variant effects on nitroglycerin efficacy. With knowledge of this genetic information, clinicians can consider either increasing the nitroglycerin dose given or choosing another vasodilator which is not metabolized by the ALDH2 enzyme, such as nitroprusside.

## Conclusions

With a focus on precision medicine and the high prevalence of the ALDH2*2 variant within the East Asian population, clinicians should consider whether a patient has this variant which increases the risk for specific diseases such as esophageal cancer and limits the metabolism of specific drugs such as nitroglycerin. One option to consider is more frequent screening for esophageal cancer for those with both the ALDH2*2 variant and a history of smoking cigarettes and/or consuming alcohol. When the potential influence of the ALDH2*2 variant is taken into consideration, relative to disease risk and drug metabolism, patients with the ALDH2*2 variant can begin receiving personalized medical management which will become standard practice in the precision medicine era.
